# Passive Fire Protection of *Taeda pine* Wood by Using Starch-Based Surface Coatings

**DOI:** 10.3390/polym13213841

**Published:** 2021-11-06

**Authors:** Svetlana Tretsiakova-McNally, Adeline Le Douarin, Paul Joseph, Malavika Arun

**Affiliations:** 1 Belfast School of Architecture and the Built Environment, Ulster University, Newtownabbey BT37 0QB, Northern Ireland, UK; ledouarinadeline@yahoo.fr; 2 Institute for Sustainable Industries and Liveable Cities, Victoria University, P.O. Box 14428, Melbourne, VIC 8001, Australia; Paul.Joseph@vu.edu.au (P.J.); Malavika.Arun@vu.edu.au (M.A.)

**Keywords:** wood substrates, starch-based colloids, surface coatings, combustion attributes, passive fire protection

## Abstract

The present paper reports the preliminary results relating to the development, subsequent application, and testing of environmentally benign starch-based formulations for passive fire protection of wood substrates. This study evaluated the effectiveness of starch colloid coatings applied onto the wood surface with a view to improving its performance when exposed to the external heat flux (35 kW/m^2^) during cone calorimetric tests. The formulations were prepared from aqueous colloid solutions of either starch alone, or in combination with inorganic salts, such as: sodium carbonate, Na_2_CO_3_, potassium carbonate, K_2_CO_3_, and diammonium hydrogen phosphate, (NH_4_)_2_HPO_4_. The fire performance of *Taeda pine* wood samples, where their top surfaces were treated with these formulations, was compared with the control sample. The thermal and combustion characteristics of the tested samples were determined with the aid of thermo-gravimetric analysis (TGA), bomb and cone calorimetric techniques, and a steady state tube furnace coupled to an FT-IR spectrometer. A significant boost of fire protection was observed when starch formulations with added inorganic salts were applied onto the wood surfaces, compared with the control sample. For example, the presence of K_2_CO_3_ in starch colloid solutions resulted in a notable delay of the ignition and exhibited a reduction in the heat release parameters in comparison with the untreated wood substrate.

## 1. Introduction

Fire safety of new and existing buildings is highly dependent on the structural elements that might be susceptible to ignition, combustion, and fire spread if/when exposed to an external heat source. Ligno-cellulosic materials, such as wood, occupy a high-level hierarchy among the natural materials, especially in the context of lightweight and ‘green’ construction methodologies [1]. Owing to a near-carbon neutrality and a unique combination of aesthetic and physical properties, wood is a popular and sustainable building material in many countries around the world. It frequently forms an integral part of the building’s exterior and interior woodworks, and is also the main source of furniture found inside our homes, schools, and offices. However, wood-based construction materials inevitably suffer from a major drawback, i.e., a relatively high level of flammability. In addition, the toxicity and the associated hazardous nature of fire-retardant formulations that are currently applied to protect wood-based substrates can pose serious problems [2]. Although the use of wood in the construction was restricted by a number of prescriptive regulations and safety requirements, the demand for timber elements is on the increase in recent years. Environmental and health implications have led to the growing demand for environmentally friendly fire retardants (FRs), as the alternatives to halogenated formulations. Therefore, it is highly prudent to devise novel means and methods of utilizing environmentally benign, non-toxic, and sustainable formulations as passive fire protective agents for wood [3].

Many fire-retardant (FR) formulations for wood substrates were developed in the last two decades, and are well-documented in the literature [2,3]. These FRs have different pathways to interfere with the main stages of thermal decomposition and combustion of wood-containing products [3]. Generally, they function in various modes such as by inhibiting or delaying the ignition, through the formation of chars and thermal insulation layers on the surfaces exposed to fire, by diluting flammable volatile mixtures, by absorbing heat through endothermic processes and by reducing heat release rates, etc. Several means for protecting wood from the impact of fires are currently used in the industry, including an impregnation through vacuum-pressure or plasma treatment methods and surface coatings [4]. The surface coatings, for example, with the intumescent systems, are attractive fireproofing solutions as they can be easily applied on to the existing, or fresh, timber elements, using paint brushes and sprayers [5]. It is predicted that in the next three years the global market of fireproof coatings for wood is expected to grow by more than 5% [6].

Despite the progress made in the last decade, there is a notable shortage of effective FR systems that can meet the growing demand for wood-based construction products. The largely heterogeneous character and the hydrophilic nature of wood are the major challenges on route to design efficient and durable FR solutions. Moreover, very little progress has been made in the wood treatments that can exhibit synergistic effects, for instance between P- and N-containing organic compounds [2]. Other synergistic components include inorganic salts (carbonates, sulfates, phosphates, etc.) that can be simply mixed with FR coatings to increase their fire performance in protecting wood surfaces. They are considered to be environmentally benign and less toxic compared with some of the common halogenated formulations. For instance, potassium carbonate, K_2_CO_3_, and a mixture of potassium carbonate, K_2_CO_3_, with urea, CO(NH_2_)_2_, in aqueous solutions were proposed as the FR agents for treatment of *Scots pine* wood [7]. Diammonium hydrogen phosphate, (NH_4_)_2_HPO_4_, commonly used as an active ingredient of fire-retarding products to combat wildfires, can lower the combustion temperature of the protected materials, increase the char residue production, and decrease the weight loss rates [8]. More recently, the 3 wt.% aqueous solutions of (NH_4_)_2_HPO_4_, K_2_HPO_4_, NH_4_Cl, (NH_4_)_2_SO_4_ and their mixtures were impregnated on to the *Oriental beech* wood [9]. It was found that the mixture of (NH_4_)_2_HPO_4_ and K_2_HPO_4_ significantly improved thermal stability of the protected wood [9]. The main disadvantage of using these salts is their high solubility in water, resulting in an increased likelihood of leaching, migrating, and crystallizing of these compounds on the surfaces of wood items [10]. Another limitation of the aqueous solutions is their poor adherence to and a potential run-off from the vertical surfaces, which can be solved by adding thickening, or gel-forming, agents such as sodium bentonite, or starch, mixed with clays [11]. Starch was also selected as a main component of FR systems in several other studies due to its good adhesion to the wood surfaces [12,13,14,15,16,17]. In addition, starch is a bio-based polymeric component derived from renewable and widely available biomass resources; it can be easily and cheaply extracted from unwanted residues such as food or agricultural waste (e.g., potato peels or corn cobs) [18]. These FRs have more advantages over halogenated or synthetic counterparts in terms of their inherent ability to produce thermally stable char residues when exposed to fires. However, the challenge of using bio-based FRs to trigger the specific flame-retardants mechanisms remains [14,19].

The main aim of this study was to reduce the flammability of wood by application of starch-based FRs onto the surfaces of *Taeda pine* substrates. The assessment of their performance was carried with the range of analytical techniques, including thermo-gravimetric analysis (TGA), bomb and cone calorimetries, and a steady state tube furnace coupled with a Fourier-transform infrared (FT-IR) spectrometer.

## 2. Materials and Methods

The planks of *Taeda pine* softwood (ML Panel, Manningtree, UK), without any visible defects, were conditioned for at least one week at a temperature of 23 ± 2 °C and at a relative humidity of 50 ± 5%. Before the application of the formulations, these boards were cut to the size of 100 mm × 100 mm × 20 mm as per requirements of the ISO 5660-1:2015 standard [20]. The moisture content (MC) was determined in conformance to the standard: BS EN 14774-2:2009 and averaged over six measurements in each case [21]. The MC for untreated wood was found to be 10.3 ± 0.3 wt.%, whereas for the samples of wood treated with starch-based formulations the MC values ranged from 13.1 to 13.5 (± 0.5 wt.%). The average density (six measurements) of the wood samples used in this study was found to be 600 ± 10 kg/m^3^.

Potato starch, anhydrous sodium carbonate, Na_2_CO_3_, and potassium carbonate, K_2_CO_3_, were purchased from Fisher Scientific (Loughborough, UK), and diammonium hydrogen phosphate, (NH_4_)_2_HPO_4_, was obtained from Sigma Aldrich (Dorset, UK). All chemicals, in their powdered forms, were used as received without any further purification.

**Figure 1:**
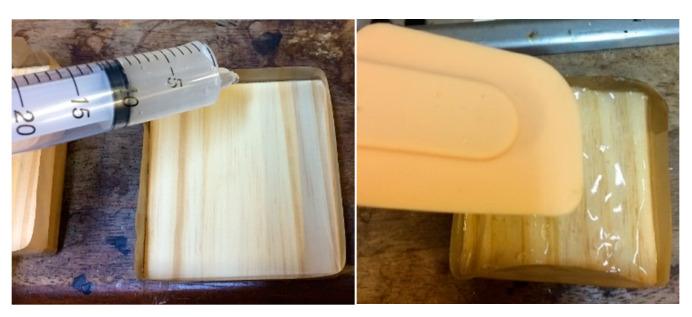
The application of starch-based coatings onto the surface of *Taeda pine* wood samples.

The FR formulations were prepared, initially, by mixing 10 g of potato starch with 100 cm^3^ of distilled water, or with 100 cm^3^ of 1.7 wt.% aqueous solutions of the inorganic salts, as the case may be, using a magnetic stirrer/hotplate at room temperature, followed by heating the mixtures to 60 °C. At this temperature, the stirring was continued (for ca. 5 min) until the uniform colloid solutions were formed. These formulations were then left to cool at room temperature for 20 min before applying them onto the wood surface. All the sides of the square wood block were protected with a brown sticky tape to ensure that only a top surface is treated. The weights of the samples were measured before and after the coating application. The equal amounts (*ca.* 10 g) of the prepared formulations were applied uniformly onto the surface of the wood substrates with the aid of a syringe and a silicon spatula (Figure 1). The coatings were allowed to dry at room temperature for 3.5 h, in a fume hood, under the ventilation with the air flux of 0.030 m^3^/s.

An IKA C200 bomb calorimeter (UK) was used to measure the heats of combustion (i.e., gross calorific values) according to the BS EN ISO 18125:2017 [22]. The samples (ca. 0.500 g) were placed into a quartz crucible with a cotton thread at its bottom in order to trigger the ignition. The samples of the top surface treated with a coating were carefully cut from the wooden block; they were not pressed into pellets to avoid any coating disruption. Then, the crucible was put into a stainless-steel vessel (a ‘bomb’) containing a small amount (5 cm^3^) of distilled water. The ‘bomb’ was filled with the oxygen up to 3.0 MPa of pressure. The sample was subsequently ignited, and the gross calorific values were recorded and displayed automatically by the instrument. These measurements were performed three times or until the variation between the obtained values was within 400 J/g.

Thermo-gravimetric analysis (TGA) runs were carried out on ca. 5–10 mg samples, on a Mettler Toledo TGA/SDTA 851^e^ instrument in the temperature range of 30–800 °C, under both nitrogen and air atmospheres, at a heating rate of 10 °C/min as per general principles described in BS EN ISO 11358-1: 2014 [23]. The specimens were taken from treated and untreated wood surfaces. 

The flammability characteristics of unprotected and protected wood samples were evaluated by employing a cone calorimeter (Dark Star Research Ltd., Nr Wrexham, UK), at the external heat flux of 35 kW/m^2^, according to the ISO 5660-1 standard [20]. As per the requirements of the standard [20], all surfaces of the samples, except for the top one, were wrapped with aluminium foil. The cone calorimeter testing was carried out in the ambient atmosphere with the flow rate in the exhaust hood of 0.0242 m^3^/s. The tests were done with a piloted ignition (a 10 kV spark igniter). The samples of wood inside a special sample holder, as described previously [24], were placed below the cone heater with the shutter on. The distance between the heater and the top surface of the sample was standard, 25 mm [20]. When the spark igniter was positioned above the sample, the shutter was simultaneously opened, and simultaneously the data collection began. The emitted gases were collected through a ventilation system. The measurements were repeated and averaged for each measured parameter.

The analyses of evolved gaseous products of the wood thermal decomposition, mainly carbon monoxide, CO, and carbon dioxide, CO_2_, were carried out using a steady state tube furnace coupled with an FT-IR spectrometer. Initially, the sample (*ca.* 1 g) was weighed out in a crucible, and then placed inside the tube furnace. A constant rate of nitrogen flow, 5 L/min, was provided via a heated transfer line fitted with an integrated soot filter (Hillesheim Gmbh, Waghausel, Germany) kept at 170 °C. The tests were run at 350 and 650 °C. The data was recorded for a duration of 30 min in each case. The gas analyses were performed on a Bruker Tensor 27 FT-IR spectrometer, equipped with a mercuric cadmium telluride detector, cooled with liquid nitrogen flowing at a rate of 6 L/min. A two-meter-long gas cell (with the volume of 2.9 L) heated to 180 °C and supplied by Infrared Analysis (model M-4-10-H) was used. The FT-IR spectral data was recorded and analysed with the aid of OPUS Data Collection software (CHROM pack) following the requirements of the ISO 19702:2015 standard [25]. The resolution of the FT-IR spectra was set to 0.5 cm^−1^.

The solid-state NMR (^31^P with CP/MAS mode) spectrum of the char residue was obtained by employing a 500 MHz Bruker machine at ambient probe conditions, typically at 10 kHz rotor speed, and the signals were calibrated against phosphoric acid as the external calibrant. The raw data were then processed by using a proprietary software from the manufacturer (TopSpin 4.0.6) [26,27].

## 3. Results and Discussion

**Figure 2:**
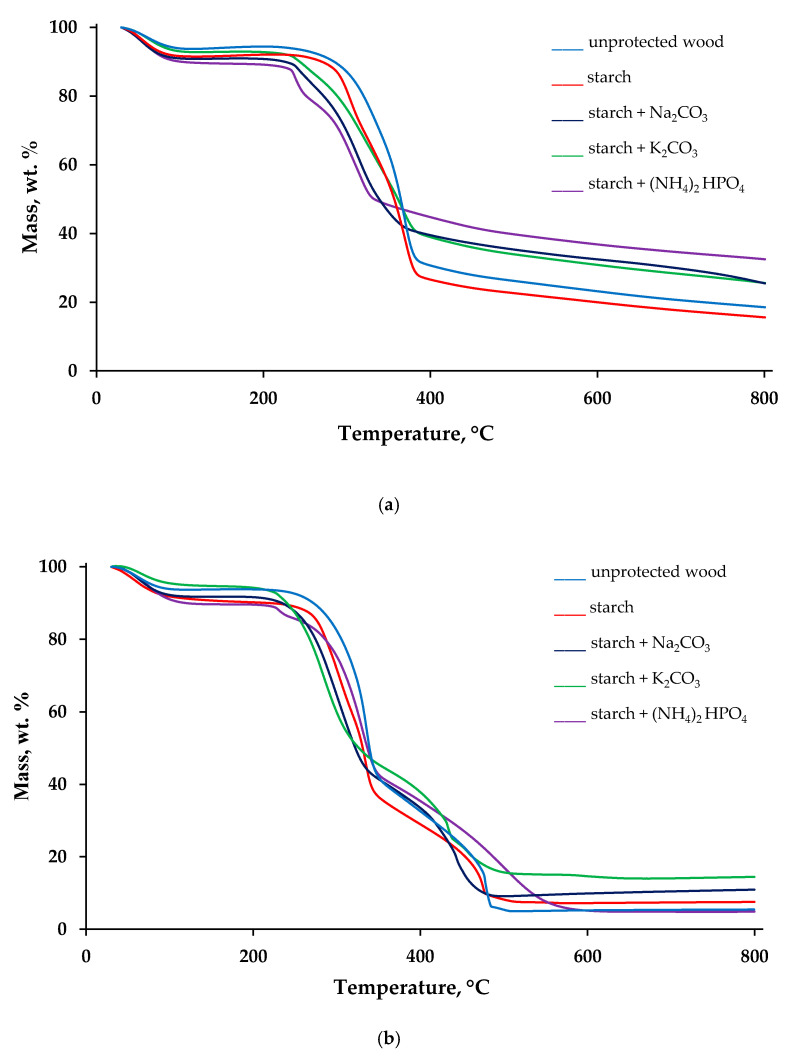
TG traces recorded on the samples of unprotected wood and wood protected with starch-based formulations, under nitrogen (**a**) and air (**b**).

Thermal decomposition behaviours of the virgin wood and the wood surfaces with applied FR formulations were evaluated, utilising a TGA technique, under nitrogen and air atmospheres. The obtained thermogravimetric (TG) traces are given in Figure 2, whereas the measured TG parameters are summarized in Table S1, given in the supplementary information section.

Figure 2a,b shows the plots of the mass (in wt.%) as a function of temperature (in °C) recorded in inert and air atmospheres. Several stages of mass loss can be observed on the TG curves obtained in nitrogen (Figure 2a). The first step of the mass loss, related to the removal of physically bound moisture, occurred in the interval from around 50 to 120 °C. The second step, which we have considered as the main thermal degradation step, commenced at 200–240 °C, depending on the starch-containing formulations. The third step, closely followed and overlapped with the second one, was finished at around 380–450 °C. The last mass loss started at 470 °C and continued until 800 °C, the end test temperature. The TG curves obtained under the air atmosphere have similar regions (Figure 2b). However, the step in the temperature region between 300 and 400 °C was found to be consisted of two stages. The lowest temperature for the onset of thermal degradation in the air was registered for the wood sample coated with ‘starch + K_2_CO_3_’ formulation (196 °C), whereas the highest value (235 °C) was recorded on the untreated wood sample. The data presented in Table S1 showed that the main loss of mass commenced 20–40 °C earlier in the case of formulations containing either Na_2_CO_3_ or K_2_CO_3_ compared with the unprotected sample. Here, it can be assumed that dissolved carbonates along with starch began to protect wood prior to its thermal degradation. Application of all the formulations also lowered the rate of mass loss, in both atmospheres. The slopes associated with the TG steps were decreased for the surfaces protected with the formulations combining both starch and inorganic salts (Figure 2).

The TG char residues obtained at 800 °C in the air atmosphere were 14–18 wt.% lower than those obtained in the inert atmosphere owing to the expected oxidation reactions. The only exception was the behaviour of the sample with ‘starch + (NH_4_)_2_HPO_4_′ formulation in the air, when the char residue was reduced slightly (4.9 wt.%) compared with the untreated wood (5.4 wt.%), which is possibly linked to the secondary oxidation and volatilisation processes. The ^31^P NMR spectrum of the solid char obtained after decomposition of wood coated with ‘starch + (NH_4_)_2_HPO_4_′ formulation peaked at δ = −1.2 ppm with a shoulder at δ = −11.7 ppm, indicative of phosphorus acid species, that could involve condensed oligomeric species (i.e., Lewis acid moieties that could aid dehydration and a char formation: see Figure S1 in the Supplementary Materials). The residue production trends confirmed that K_2_CO_3_ and (NH_4_)_2_HPO_4_ are the most efficient additives to starch colloid solutions that are capable to promote char layer formation on the wood surfaces. Indeed, the char residue obtained at 800 °C in nitrogen grew by 14 wt.% when the formulation ‘starch + (NH_4_)_2_HPO_4_’ was applied; whilst in the air, this growth was more 9 wt.% for the surfaces coated with ‘starch + K_2_CO_3_’ (Table S1). 

**Figure 3:**
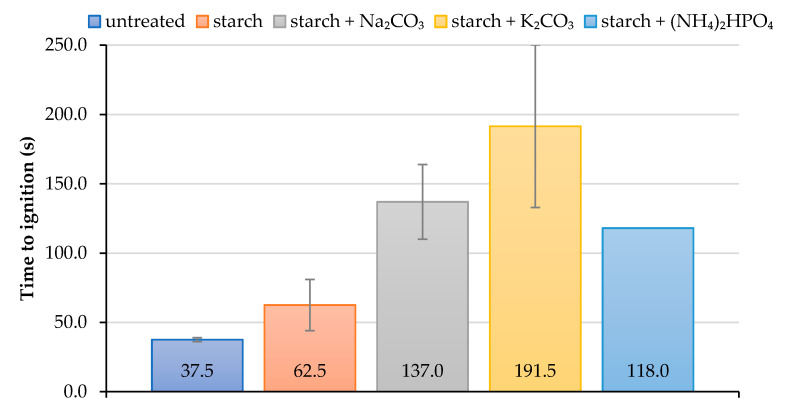
The average values of time to ignition for unprotected and protected wood samples.

As it follows from cone calorimetry tests, the formulations containing starch with added inorganic salts delayed the ignition of wood surface for much longer compared with the coating based on the starch alone (Figure 3). The application of the starch-based formulations, particularly those with salts, onto the wood surfaces significantly delayed their ignition. For example, ‘starch + K_2_CO_3_’ coating increased the time to ignition more than 5-fold compared with the virgin wood. It should be noted that the coating containing starch with added K_2_CO_3_ made the ignition of the wood extremely difficult to achieve during cone calorimetry testing; this might have led to the lower repeatability of the measured values. Indeed, the measurements error was the highest for this type of formulation, whilst for the coating with diammonium hydrogen phosphate it was found to be zero (the error bar is not visible) (Figure 3).

The average values of the parameters that characterise flammability and heat transfer processes in the untreated and treated wood were obtained with the aid of cone calorimetry for the samples exposed to 35 kW/m^2^ heat flux (Table 1 and Table 2). 

**Figure 4:**
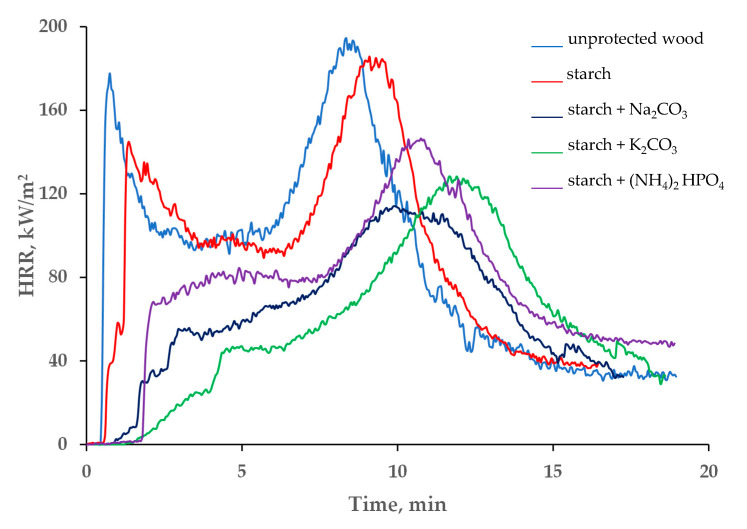
The HRR vs. time profiles of unprotected wood and the wood coated with starch-based formulations.

The variations in the heat release rates (HRR), and especially in the peak of the heat release rate (pHRR) and the total heat release (THR), can quantitatively demonstrate the fire hazards for a particular material; generally, the higher the values of the HRRs the higher the intensity of fire involving this material [28]. The data presented in Table 1 and in Figure 4 indicate that the HRR was reduced significantly after treatment of the *Taeda pine* surface with the studied formulations. The first peak, associated with the ignition, was either drastically reduced or completely disappeared from the curves HRR vs. time (Figure 4). This effect was particularly pronounced in the systems containing salts dissolved in colloid solutions. Approximately 2 min after the exposure to heat, the inorganic salts started to decompose by absorbing the heat through endothermic process(es), which resulted in slowing down the flaming combustion and reducing the HRR values. Compared with the untreated wood surface, the average heat release rate was dropped by 40.6 kW/m^2^ for the ‘starch + Na_2_CO_3_’ and by 42.3 kW/m^2^ for the ‘starch + K_2_CO_3_’ coatings, whist the pHRR values were reduced by 51.0 and 57.4 kW/m^2^, respectively. In addition, compared with the virgin wood, the pHRR was shifted to higher times, by approximately 2–4 min, when the coatings containing starch with sodium or potassium carbonates were applied onto the wood surface (Figure 4). These formulations were able to keep the HRR relatively low (below 120 kW/m^2^) for longer periods of time compared with the unprotected wood or to the wood with the ‘starch’ coating.

A notable reduction in the average values of the THR were also observed for the systems with added carbonate salts (Table 1). For example, application of the ‘starch + Na_2_CO_3_’ resulted in a 25.3 MJ/m^2^ drop of the THR, compared with the untreated wood. The average mass loss rate (MLR), its peak values and the total mass loss were also lower for the wood surfaces with the applied formulations (Table 1 and Figure S2). Once again, the best protection against the mass loss was achieved by adding potassium carbonate to the starch colloid solution: the average MLR was the lowest among all the tested formulations 5.1 × 10^−2^ g/s.

**Figure 5:**
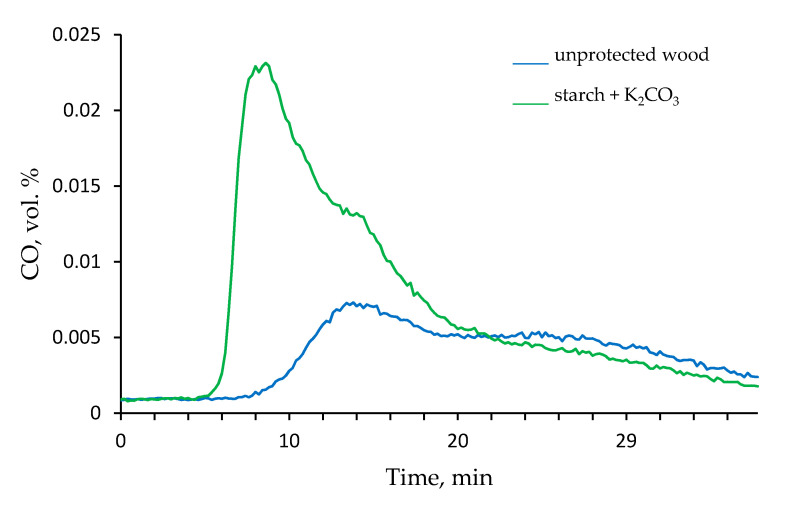
The time dependence of the amount of CO produced by unprotected wood and by the wood coated with ‘starch + K_2_CO_3_’ formulation in the tube furnace (350 °C, nitrogen atmosphere).

The total smoke produced during cone calorimetry testing was decreased significantly in the case of samples with the applied formulations as shown in Table 2. For example, the total smoke released dropped by almost 50%, whereas the peak of rate smoke production (RSP) was reduced by a factor of 1.6 for the wood sample protected with the colloid solution of starch mixed with Na_2_CO_3_. Similar values were recorded for the wood treated with the ‘starch + K_2_CO_3_’ formulation. However, this coating resulted in a higher value of total CO volume and in the increased CO/CO_2_ ratio as opposed to the virgin wood (Table 2). This was also noted at 350 °C in the tube furnace-FT-IR measurements that will be discussed later (Figure 5). It is likely that the decomposition of carbonates inhibited the ignition and combustion of wood surfaces. This is clearly evidenced from the values of effective heat of combustion given in Table 2. The undesirable increase in the volume of CO, particularly at the early stages of the wood exposure to the radiant heat, could be tackled by replacing K_2_CO_3_ with other salts, for example with Na_2_CO_3_ or by using starch alone. Interestingly, the total volume of CO_2_, which may act as a diluent and/or as a blowing agent, was lower for the coatings containing both carbonates compared with the unprotected wood surface as measured through cone calorimetry (Table 2).

**Figure 6:**
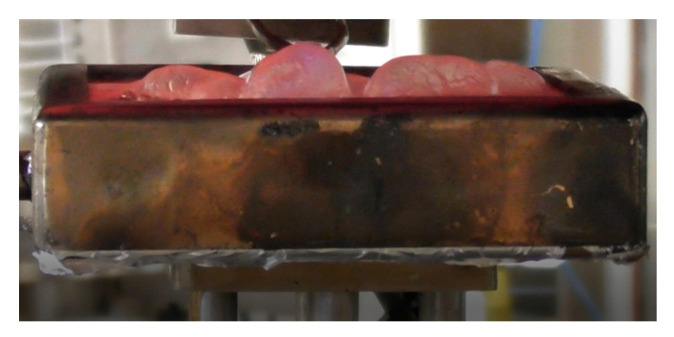
The behaviour of the coating based on starch acting as a blowing agent, heat flux 35 kW/m^2^, 20 s before the ignition.

**Figure 7:**
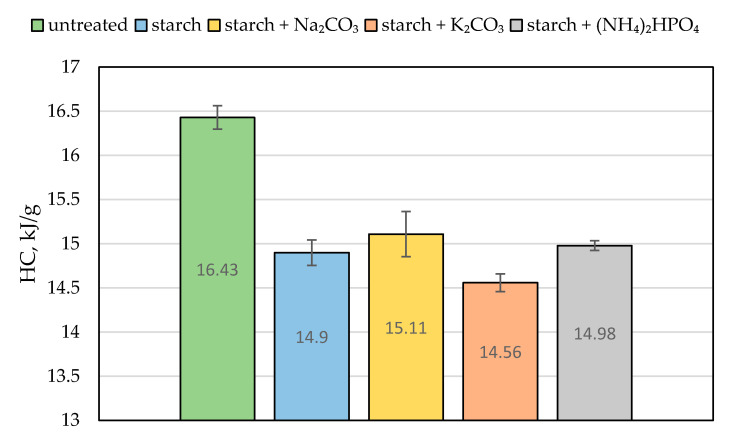
The average heat of combustion (HC, in kJ/g) for the untreated wood and samples coated with starch-based formulations.

The value of the effective heat of combustion (EHC) can be considered as another quantitative characteristic of the material’s combustion attribute. It expresses the amount of energy released by a unit of mass of the burning wood and is evaluated from the ratio of HRR to MLR [5]. Compared with the virgin material, the ‘starch + K_2_CO_3_’ formulation reduced the EHC by a factor of 1.8. The similar drop in the EHC value was observed for the coating containing sodium carbonate (Table 2). We assume that the endothermic decomposition of carbonates, which liberated carbon dioxide, partially removed the heat from the wood surface and reduced its temperature. This effect is clearly visible in Figure 6, showing a blowing action of starch coating 20 s prior to ignition. The release of carbon dioxide not only diluted the mixture of flammable volatiles formed above the heated wood surface but also provided a cooling effect [7]. This trend was also confirmed by the bomb calorimetry measurements: the average values of the heat of combustion (HC) were lower for the protected wood than for the untreated material (Figure 7). Similar trends were reported elsewhere for the impregnation of wood samples with formulations containing potassium carbonate that resulted in longer times to ignition, reduced heat release rates, and lower values of heat of combustion [7,9]. Moreover, the snapshots taken from the video recordings of the cone calorimetry tests demonstrated that the protective coating containing K_2_CO_3_ made the flaming combustion of wood difficult to achieve (Figure S3), as opposed to the untreated surface.

Thus, the ‘starch + K_2_CO_3_’ formulation demonstrated the maximum influence on the flammability and associated combustion parameters, and therefore was selected for the steady state tube furnace testing at 350 and 650 °C. Figure 5 shows the time profiles of the CO volumetric percentage released by the untreated wood and the wood treated with ‘starch + K_2_CO_3_’ coating. It is evident that in the latter case, the volumetric concentration of CO produced was significantly increased in 9 min since the start of tube furnace testing. Interestingly, this difference between the unprotected and protected wood samples became almost negligible when the temperature in the tube furnace was set at 650 °C. As for the release of CO_2_, only small concentrations, which did not vary with time, were registered at 350 °C. However, at 650 °C the percentage of CO_2_ detected at around 20 min, was almost doubled when the wood was coated with the ‘starch + K_2_CO_3_’ formulation. Figures S4 and S5 (see Supplementary Materials section) show the FT-IR spectra recorded at 350 and 650 °C, at around 1 min 15 s, since the insertion of the samples into the tube furnace. The absorbance peaks observed at 2400 cm^−1^ are assigned to CO_2_ and the signals in the interval from 2250 and 2000 cm^−1^ are related to the CO released in the tube furnace. The intensities of peaks associated with CO and CO_2_ in the FT-IR spectra were significantly higher for the wood with applied ‘starch + K_2_CO_3_’ formulation. 

## 4. Conclusions

This study presented the initial results of the wider research attempting to utilize the biomass-based materials to produce effective FRs. The starch-based formulations were prepared by simple mixing of inexpensive and widely available components: water, starch and inorganic salts. The preparation of the formulations was done at room temperature (except for a very short period of heating to obtain colloid starch solutions) and atmospheric pressure. Another advantage is in the simplicity of the equipment used to apply the coatings onto wood surfaces. The main findings are summarised below:

- Starch-based coatings are found to be very effective and suitable for passive fire protection of wood-based substrates. The values of the measured parameters pertaining to the combustion behaviours of the tested samples clearly demonstrated the overall efficacy of the starch-based coatings, particularly those with the added inorganic salts. This was evident from the ignition delay, the increased char yields, the reduced heats of combustion, slower rates of mass loss for the wood samples with applied coatings as opposed to the unprotected wood. The application of these formulations resulted in a significant reduction in the heat release parameters, pHRR, HRR, and THR.
- The coating based on the colloidal starch formulation with diammonium hydrogen phosphate (NH_4_)_2_HPO_4_ demonstrated very good char promoting attributes. The ^31^P NMR data indicated the phosphorus acid species that may promote the processes of dehydration and char formation.
- A significant boost in fire protection of wood coatings was achieved upon the addition of sodium carbonate, Na_2_CO_3_, and potassium carbonate, K_2_CO_3_, to the starch colloid solutions. The ‘starch + K_2_CO_3_’ formulation demonstrated the maximum influence on the ignition and flammability parameters, whereas application of the ‘starch + Na_2_CO_3_’ reduced volume of toxic gases and smoke produced. The exact mechanism underpinning the action of these formulations is not yet identified and requires further investigation, perhaps through the use of hyphenated techniques, such as TGA-GC/MS, pyrolysis-GC/MS, etc. It is likely that carbon dioxide CO_2_ generated upon the endothermic decomposition of carbonates acts both as a diluent and a blowing agent.

Given the availability, low costs, and sustainable nature of these admixtures, they warrant further investigations of the potential use of starch colloids as the effective protective coatings for the wood substrates. It is also highly relevant to note here that wood and its products (often termed as ‘engineered’ wood) are increasingly being used in the construction sector, especially for high-rise buildings. Therefore, the desirable outcome from the present work is likely to open up the possibilities of deploying wood as a versatile material, with a wider applicability, that is also coupled with improved fire safety. Hence, the the impact of such approach is likely to resonate beyond the academic domain, for example, in the commercial exploration of widely available, non-toxic, and bio-sourced compounds for formulating protective surface coatings, for safe and resilient construction materials. This would aid the transformation of the modern construction industry through sustainable practices for years to come.

## Figures and Tables

**Table 1:** The averages of THR, HRR, and MLR and the corresponding peak values measured in the standard cone calorimeter at 35 kW/m^2^.

Formulation	Peak HRR (kW/m^2^)	THR (MJ/m^2^)	HRR (kW/m^2^)	Peak MLR × 10^2^ (g/s)	Mass Loss (%)	MLR × 10^2^ (g/s)
Untreated	187.2	89.8	116.7	11.5	79.8	7.1
Starch	187.7	91.0	106.1	16.0	78.3	7.6
Starch + Na_2_CO_3_	136.2	64.6	76.10	9.50	73.0	5.5
Starch + K_2_CO_3_	129.8	65.1	74.40	9.50	73.8	5.1
Starch + (NH_4_)_2_HPO_4_	148.0	87.1	86.40	9.00	79.1	5.2

**Table 2:** The average values of SEA, EHC, total smoke, total CO and CO_2_, and their ratio measured in the standard cone calorimeter at 35 kW/m^2^.

Formulation	Total Smoke (m^2^)	SEA (m^2^/g)	Total CO × 10^4^ (m^3^)	Total CO_2_ × 10^3^ (m^3^)	Ratio CO/CO_2_	EHC (kJ/g)
Untreated	4.39	9.19	11.22	19.24	5.83	29.2
Starch	3.08	5.67	9.02	17.24	5.23	36.4
Starch + Na_2_CO_3_	2.36	3.54	11.83	12.00	9.85	15.5
Starch + K_2_CO_3_	2.89	5.46	15.63	10.75	14.5	16.0
Starch + (NH_4_)_2_HPO_4_	3.11	5.38	14.43	15.76	9.16	21.8

## Data Availability

Data is contained within the current article and in the Supplementary Materials to this article (https://www.mdpi.com/article/10.3390/polym13213841/s1).

## Supplementary Materials

The following are available online at https://www.mdpi.com/article/10.3390/polym13213841/s1, Figure S1: Solid-state ^31^P NMR spectrum of char obtained from wood coated with ‘starch + (NH_4_)_2_HPO_4_’ formulation (the abscissa denotes the chemical shift values, δ, in ppm, and the ordinate corresponds to the signal intensity in arbitrary units, Figure S2: The MLR vs. time profiles of unprotected wood and the wood surfaces coated with starch + inorganic salt formulations, Figure S3: The snapshots taken from the videos recorded during cone calorimetry testing, 10 s after the ignition: (a) untreated wood; (b) wood coated with ‘starch + K_2_CO_3_’ formulation, Figure S4: FT-IR spectra of gaseous products produced in the tube furnace during decomposition of untreated wood (black) and wood coated with ‘starch + K_2_CO_3_’ formulation (green), 350 °C, nitrogen atmosphere (the abscissa is wavenumber (in cm^−1^) and the ordinate is absorbance (in arbitrary units, Figure S5: FT-IR spectra of gaseous products produced in the tube furnace during decomposition of untreated wood (black) and wood coated with ‘starch + K_2_CO_3_’ formulation (yellow), 650 °C, nitrogen atmosphere (the abscissa is wavenumber (in cm^−1^) and the ordinate is absorbance (in arbitrary units), Table S1: The onset temperature (T_onset_) of the main degradation step, and char residues at 800 ^o^C measured trough TGA in nitrogen and in air atmospheres.
