# Supplementary material for: Passive Fire Protection of Taeda pine Wood by Using Starch-Based Surface Coatings
Source: Polymers (Basel). 2021 Nov 6;13(21):3841. doi: 10.3390/polym13213841 (PMC8587060; doi:10.3390/polym13213841)
Supplement: Supplementary file 1 [file polymers-13-03841-s001.zip › ATMcALDPJMA_Supplementary materials171021.pdf]

polymers MDPI

Table S1: The onset temperature (T_{onset}) of the main degradation step, and char residues at 800 °C measured trough TGA in nitrogen and in air atmospheres.

Formulation	T_{onset} in nitrogen (°C)	Char residue in nitrogen (wt. %)	T_{onset} in air (°C)	Char residue in air (wt. %)
untreated	217	18.5	235	5.40
starch	242	15.6	232	7.50
starch + Na_2CO_3	200	24.5	206	10.9
starch + K_2CO_3	200	25.9	196	14.5
starch + $(NH_4)_2HPO_4$	216	32.5	220	4.90

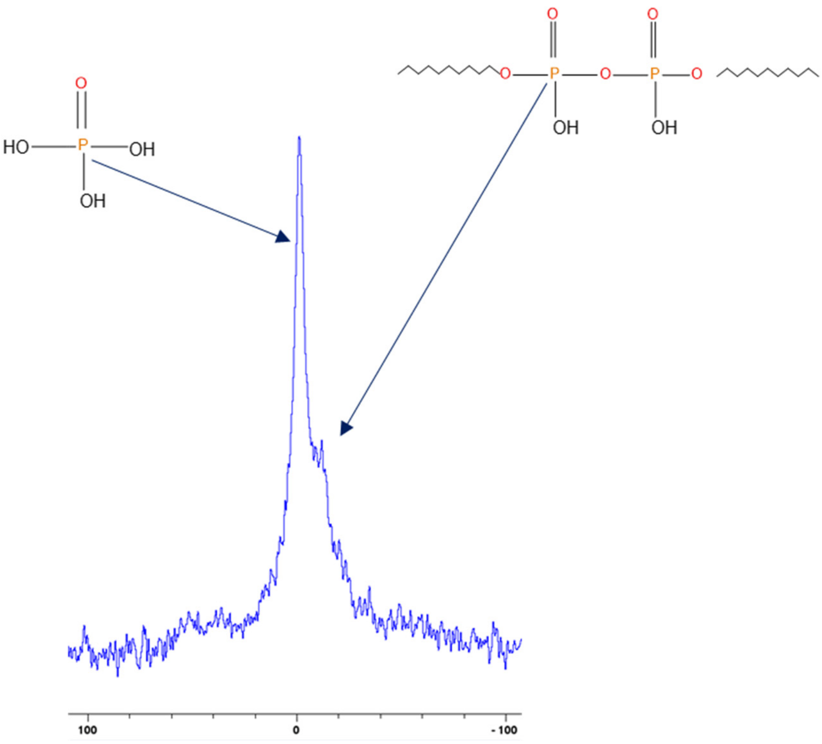

Figure S1: Solid-state ^{31}P NMR spectrum of char obtained from wood coated with 'starch + $(NH_4)_2HPO_4$' formulation (the abscissa denotes the chemical shift values, δ, in ppm, and the ordinate corresponds to the signal intensity in arbitrary units.

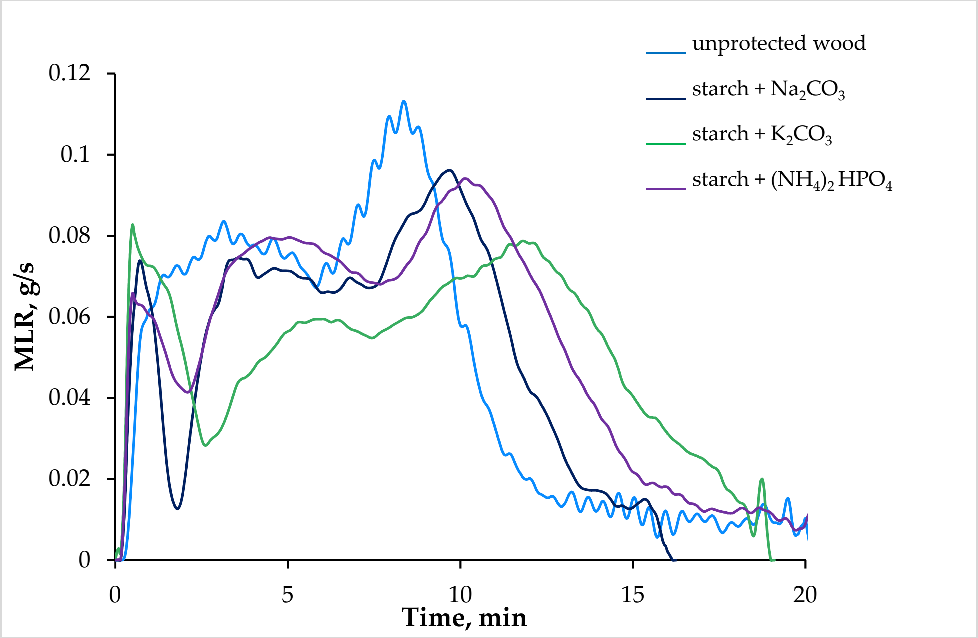

Figure S2: The MLR *vs*. time profiles of unprotected wood and the wood surfaces coated with starch + inorganic salt formulations.

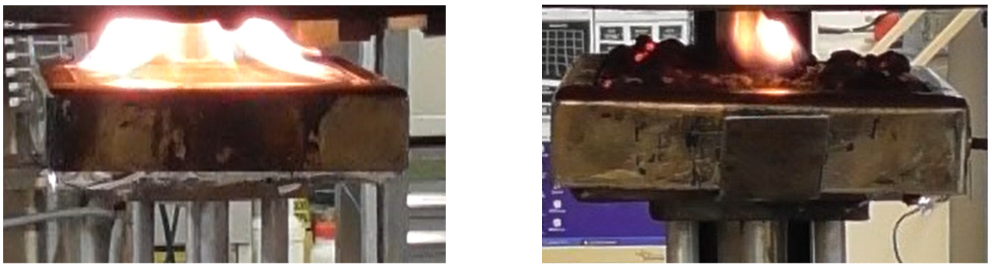

(a) (b)

Figure S3: The snapshots taken from the videos recorded during cone calorimetry testing, 10 seconds after the ignition: (a) untreated wood; (b) wood coated with 'starch + K_2CO_3' formulation.

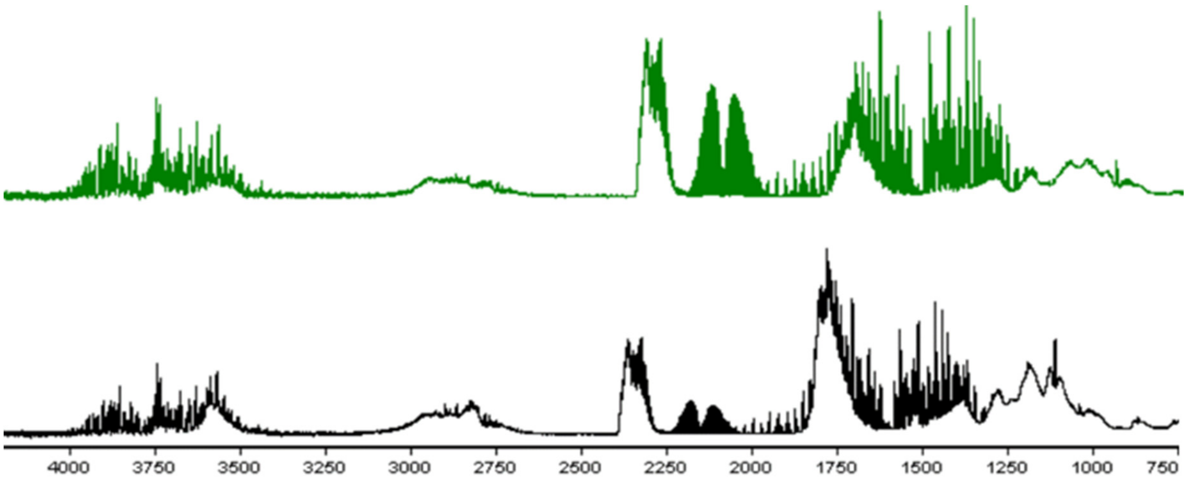

Figure S4: FT-IR spectra of gaseous products produced in the tube furnace during decomposition of untreated wood (black) and wood coated with 'starch + K_2CO_3' formulation (green), 350 °C, nitrogen atmosphere (the abscissa is wavenumber (in cm^{-1}) and the ordinate is absorbance (in arbitrary units).

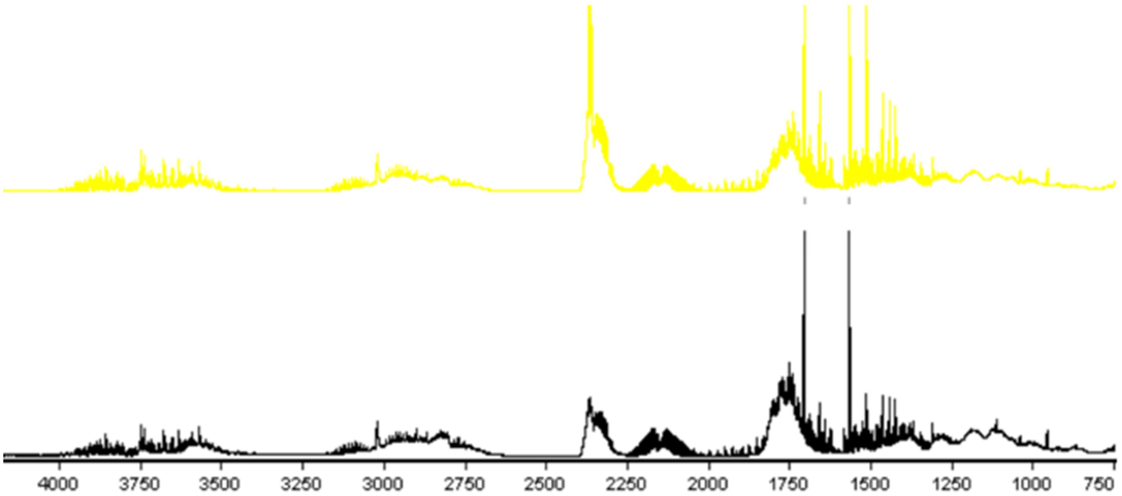

Figure S5. FT-IR spectra of gaseous products produced in the tube furnace during decomposition of untreated wood (black) and wood coated with 'starch + K_2CO_3' formulation (yellow), 650 °C, nitrogen atmosphere (the abscissa is wavenumber (in cm^{-1}) and the ordinate is absorbance (in arbitrary units).
